# Diagnostic Performance of Magnification and Ultrasonic Troughing in Detecting Second Mesiobuccal Canals: A Two-Factor Experimental Study

**DOI:** 10.7759/cureus.108940

**Published:** 2026-05-15

**Authors:** Mehmet Adiguzel, Furkan Ozeken

**Affiliations:** 1 Department of Endodontics, Faculty of Dentistry, Hatay Mustafa Kemal University, Antakya, TUR; 2 Department of Endodontics, Faculty of Dentistry, Istanbul Beykent University, Istanbul, TUR

**Keywords:** dental operating microscope, endodontics, maxillary first molar, mb2 canal, second mesiobuccal canal, ultrasonic troughing

## Abstract

Background

The detection of the second mesiobuccal (MB2) canal in maxillary first molars remains a major challenge in endodontic practice because of complex root canal anatomy and frequent calcification. This controlled experimental study evaluated the diagnostic performance of magnification and ultrasonic troughing, applied alone or in combination, for MB2 canal detection.

Methods

A total of 208 extracted human maxillary first molars were randomly allocated into four groups (n = 52 each): direct visual method (DVM) without troughing, DVM with troughing, dental operating microscope (DOM) without troughing, and DOM with troughing. A two-factor experimental design was used to assess the independent and combined effects of magnification and ultrasonic troughing. After standardized access preparation, MB2 detection was evaluated clinically and verified by root cross sections. Detection rates were compared using chi-square tests with post hoc pairwise comparisons, and multivariable binary logistic regression was performed.

Results

MB2 canal detection rates differed significantly among groups (p < 0.001). The lowest detection rate was observed with the DVM without troughing (21/52, 40.4%), whereas the highest rate was observed with the DOM with troughing (46/52, 88.5%). Intermediate detection rates were observed with troughing alone (30/52, 57.7%) and magnification alone (35/52, 67.3%). Magnification (OR = 3.90, p < 0.001) and ultrasonic troughing (OR = 2.54, p = 0.003) were independently associated with improved MB2 detection.

Conclusions

Magnification and ultrasonic troughing independently improved MB2 canal detection in maxillary first molars, with the highest diagnostic performance achieved when both techniques were combined. These findings may improve clinical decision-making during the detection of MB2 canals in routine endodontic practice.

## Introduction

The root canal configuration of maxillary molar teeth is generally complex, particularly in the mesiobuccal root, which frequently exhibits anatomical variations, additional canals, and intricate morphologies. Numerous studies have reported that the incidence of the second mesiobuccal (MB2) canal in maxillary first molars ranges from 36.3% to 97.6% [1-4], indicating that most maxillary first molars possess four canals. However, the clinical detection of the MB2 canal remains challenging and is influenced by several factors, including operator experience, treatment duration, and the diagnostic or magnification methods employed [5-7]. Accordingly, the incidence of missed canal anatomy in maxillary first molars has been reported to range between 41.3% and 46.5%, making MB2 localization one of the major challenges in contemporary endodontic practice [8-11].

Missed MB2 canals are recognized as a leading cause of endodontic failure in maxillary molars, as untreated canal spaces may harbor bacterial biofilms that contribute to persistent periapical pathology and reinfection [12,13]. Therefore, accurate localization of this canal is essential for achieving long-term success in root canal therapy. To improve canal detection and reduce the risk of missed anatomy, clinicians are encouraged to use enhanced visualization and diagnostic aids beyond conventional direct visual methods (DVMs) [14].

In addition to anatomical complexity, pulp calcification and secondary dentin deposition may further obscure the pulpal floor anatomy, particularly in narrow or curved MB2 canals, thereby complicating canal orifice identification. To overcome these limitations, ultrasonic troughing has been proposed as a minimally invasive technique involving selective dentin removal along the pulpal floor to facilitate access to calcified or hidden canal orifices. When performed in a controlled manner, troughing may improve canal visibility while preserving tooth structure [15].

The DVM has traditionally been used for canal identification; however, MB2 detection rates with this approach alone have been reported to be relatively low [16]. The introduction of the dental operating microscope (DOM) has provided improved magnification and illumination, enabling clinicians to identify accessory canals and subtle morphological landmarks that are otherwise difficult to visualize [17]. Moreover, ultrasonic instruments allow precise and conservative dentin removal during troughing, potentially enhancing access to calcified or hidden canal orifices, particularly when used under magnification.

Several studies have suggested that the use of ultrasonic instruments, particularly when combined with DOMs, facilitates the localization of the MB2 canal by enabling selective dentin removal and enhanced visualization of the pulpal floor. This combined approach has been reported to improve MB2 canal detection in both experimental and clinical settings, supporting its role as an adjunctive strategy in the management of anatomically complex maxillary molars [18].

Although several studies have investigated the effect of magnification on MB2 canal detection, many were limited by smaller sample sizes or sequential study designs [7], making it difficult to isolate the independent and combined effects of magnification and ultrasonic troughing. Furthermore, advances in optical and ultrasonic technologies over the past decade warrant a reevaluation of earlier findings using contemporary equipment and standardized protocols. In this context, a controlled two-factor experimental approach may provide a more quantitative understanding of how each variable contributes to MB2 canal detection. Therefore, this study aimed to evaluate the independent and combined effects of magnification and ultrasonic troughing on MB2 canal detection in maxillary first molars using a controlled two-factor experimental design.

## Materials and methods

Study design

This in vitro randomized experimental study was approved by the Hatay Mustafa Kemal University Non-Interventional Clinical Research Ethics Committee (approval 2025/14-43). Extracted human maxillary first molars with anonymized identifiers were used. The study aimed to evaluate the effects of the magnification method (DVM vs. DOM) and ultrasonic troughing (present vs. absent) on the detection of the MB2 canal. A minimum sample size of 52 teeth per group (total = 208) was determined by power analysis based on pilot data, providing at least 80% power at a 5% significance level.

Randomization

The teeth were randomly assigned to four groups of equal size using a computer-generated randomization sequence. All procedures were performed by a single calibrated operator, and an independent examiner blinded to the experimental groups recorded MB2 detection outcomes to minimize observer bias. The operator had extensive clinical experience in endodontic treatment and routine use of DOMs and ultrasonic instruments.

Sample selection and storage

Teeth with complete root formation, intact furcation, and no cracks, restorations, resorptions, or history of endodontic treatment were included. Teeth with caries, structural defects, resorption, or previous endodontic treatment were excluded. All samples were stored in a 0.1% thymol solution and rehydrated in distilled water for 24 hours before testing.

Grouping

Two independent variables were tested: magnification method (DVM or DOM) and troughing application (present or absent).

A standard access cavity protocol was applied to all samples, and irrigation and bur parameters were kept constant across all groups. Access cavities were prepared following a conventional endodontic access design to ensure adequate visualization of the pulpal floor and standardized access geometry across all specimens. Four experimental groups were formed as follows:

Group 1 (DVM Without Troughing, DVM-NT)

Procedures were performed under the DVM without the use of a DOM. After completion of the access cavity, MB2 canal exploration was performed using a DG16 endodontic explorer and #08-15 K-files without ultrasonic instrumentation. The search time for each sample was standardized to two minutes. Detection of the MB2 canal was defined as the stable placement of a #10 or #15 K-file into a separate canal orifice.

Group 2 (DVM With Troughing, DVM-T)

Procedures were performed under the DVM. If the MB2 canal could not be located initially, ultrasonic troughing was performed using a piezoelectric unit (D600 LED, Woodpecker, Guilin, China) and a diamond-coated endodontic ultrasonic tip (E3D) under continuous water irrigation. The operator advanced in a palato-mesial direction, removing dentin in a controlled manner up to 2 mm initially. If the MB2 canal was not identified, troughing was cautiously extended beyond 2 mm when necessary. Troughing depth was measured using a millimeter-marked periodontal probe. After troughing, MB2 detection was reevaluated and recorded.

Group 3 (DOM Without Troughing, DOM-NT)

Procedures were performed under a DOM (Leica M320, Leica Microsystems, Wetzlar, Germany). Following access preparation, MB2 exploration was carried out using a DG16 explorer and #08-15 K-files without ultrasonic instrumentation. The search time for each sample was limited to two minutes.

Group 4 (DOM With Troughing, DOM-T)

Procedures were performed under a DOM (Leica M320, Leica Microsystems). If the MB2 canal was not detected initially, ultrasonic troughing was performed using the same device and tip parameters as in Group 2. The operator advanced in a palato-mesial direction, with controlled dentin removal up to 2 mm initially. If the MB2 canal was not identified, troughing was cautiously extended beyond 2 mm when necessary. Troughing depth was measured using a millimeter-marked periodontal probe. Inspection was performed under magnification after every 0.5 mm of advancement, and the procedure was terminated upon signs of excessive dentin removal or potential perforation. After troughing, the MB2 canal was reevaluated.

Reference verification

After all procedures, the mesiobuccal root of each tooth was sectioned perpendicular to its long axis at the coronal, middle, and apical thirds. Each section was examined under a stereomicroscope to assess the presence, morphology, and configuration (separate, fused, or isthmus-connected) of the MB2 canal. This method served as the reference standard for MB2 canal identification. For each sample, MB2 canal detection was recorded as “0” (not detected) or “1” (detected). After sectioning, specimens in which an MB2 canal was anatomically absent were excluded because the outcome (detection) was not applicable. These teeth were replaced with newly selected specimens meeting the inclusion criteria to maintain the prespecified sample size, and replacements underwent the same random allocation and experimental procedures.

Statistical analysis

Statistical analysis was performed using IBM SPSS Statistics for Windows, version 26.0 (released 2018; IBM Corp., Armonk, NY, USA). MB2 canal detection rates among the experimental groups were compared using the chi-square test. When significant differences were identified, post hoc pairwise comparisons were conducted with Bonferroni adjustment. A predefined subgroup analysis including only the troughing groups was performed to compare the required troughing depth, and Fisher’s exact test was used when appropriate. To evaluate the independent effects of magnification and ultrasonic troughing on MB2 canal detection, multivariable binary logistic regression analysis was performed. ORs with corresponding p-values were calculated, and the level of statistical significance was set at p < 0.05.

## Results

**Figure 1:**
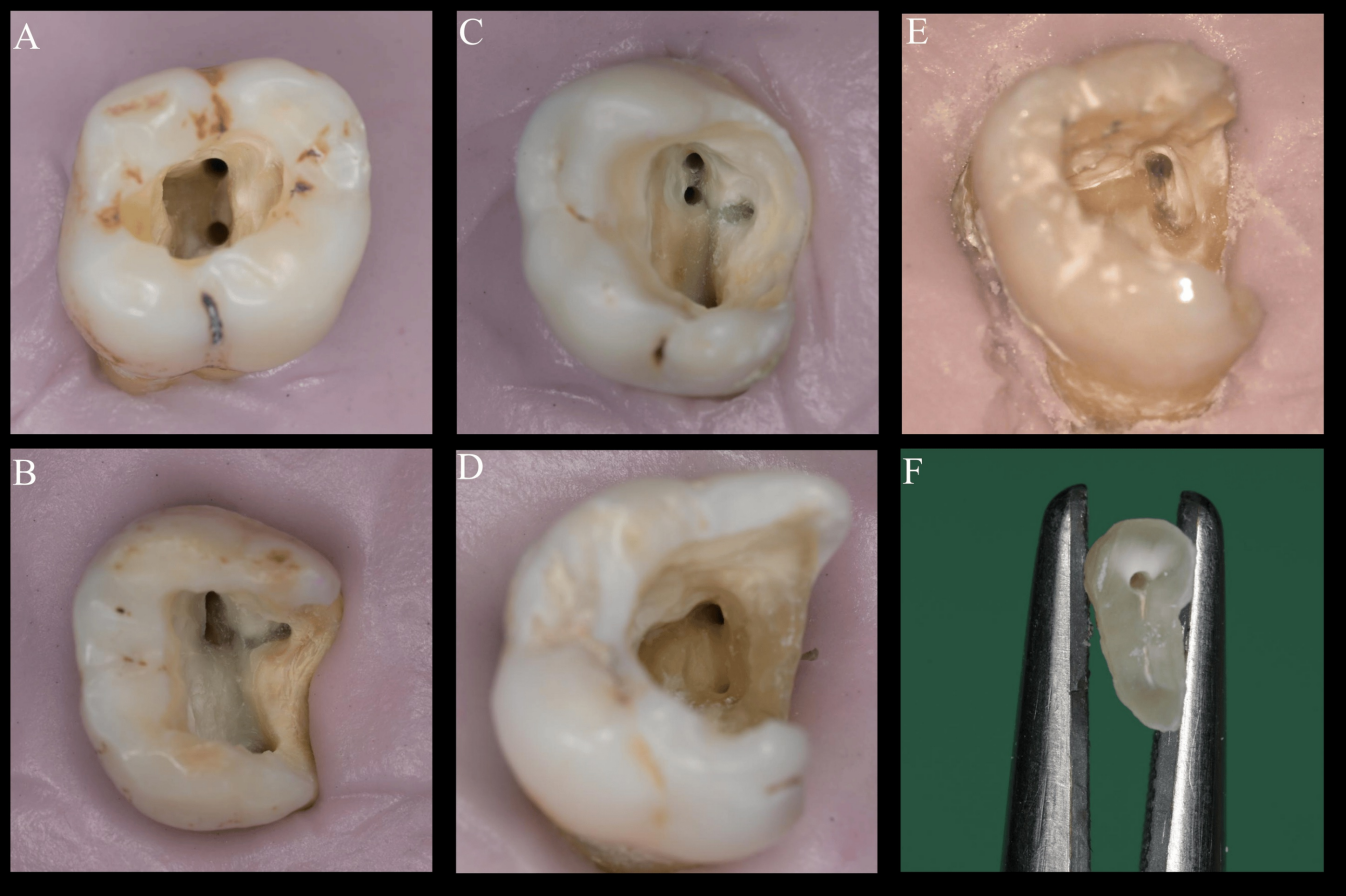
Representative images of MB2 canal detection under different experimental conditions (A) DVM-NT. (B) DVM-T. (C) DOM-NT. (D) DOM-T. (E) Example of MB2 not clinically detected. (F) Root cross section corresponding to panel E. DOM-NT, dental operating microscope without troughing; DOM-T, dental operating microscope with troughing; DVM-NT, direct visual method without troughing; DVM-T, direct visual method with troughing; MB2, second mesiobuccal

A total of 208 maxillary first molars were evaluated, with 52 specimens assigned to each experimental group. The distribution of MB2 canal detection and troughing depth is presented in Table 1, and representative images are shown in Figure 1.

**Table 1:** MB2 canal detection and troughing depth according to the examined methods Different superscript letters (ᵃ,ᵇ) indicate statistically significant differences between groups based on post hoc pairwise comparisons with Bonferroni adjustment (p < 0.05). Troughing depth was evaluated only in groups where ultrasonic troughing was performed. Overall comparison among groups: χ² = 27.04, p < 0.001. DOM-NT, dental operating microscope without troughing; DOM-T, dental operating microscope with troughing; DVM-NT, direct visual method without troughing; DVM-T, direct visual method with troughing; MB2, second mesiobuccal

Group	n	MB2 detected (n, %)	2-mm troughing (sufficient, n)	>2-mm troughing (required, n)
DVM-NT	52	21 (40.4%)ᵃ	-	-
DVM-T	52	30 (57.7%)ᵃ	22	8
DOM-NT	52	35 (67.3%)ᵃᵇ	-	-
DOM-T	52	46 (88.5%)ᵇ	40	6

MB2 canals were identified in 21/52 teeth (40.4%) in the DVM-NT group. Higher detection rates were observed in the DVM-T (30/52 teeth, 57.7%) and DOM-NT (35/52 teeth, 67.3%) groups, whereas the highest detection rate was recorded in the DOM-T group (46/52 teeth, 88.5%). The overall comparison among groups revealed a statistically significant difference (χ² = 27.04, p < 0.001).

Post hoc analyses showed that the DOM-T group differed significantly from both the DVM-NT and DVM-T groups. No statistically significant differences were detected among the remaining group comparisons.

When only the troughing groups were considered, 2-mm troughing was sufficient in 22 teeth in the DVM-T group and in 40 teeth in the DOM-T group, with a greater proportion of cases in which 2-mm troughing was sufficient in the DOM-T group. The proportion of teeth requiring troughing deeper than 2 mm did not differ significantly between these two groups (p > 0.05).

Binary logistic regression analysis (Table 2) further demonstrated that the use of magnification was independently associated with increased MB2 canal detection (OR = 3.90; p < 0.001). Ultrasonic troughing was also significantly associated with MB2 canal detection (OR = 2.54; p = 0.003).

**Table 2:** Binary logistic regression analysis of factors associated with MB2 canal detection DOM, dental operating microscope; DVM-NT, direct visual method; MB2, second mesiobuccal

Variable	OR	95% CI	p-Value
Magnification (DOM vs. DVM)	3.9	2.10-7.25	<0.001
Ultrasonic troughing (yes vs. no)	2.54	1.38-4.70	0.003

## Discussion

One of the critical determinants of successful root canal treatment is the identification and treatment of all existing root canals. Karabucak et al. [9] reported that missed canals were present in 41% of endodontically treated teeth and increased the likelihood of associated periapical lesions by 4.38-fold. Among these, the MB2 canal of maxillary molars is particularly prone to being overlooked due to its complex anatomy, narrow dimensions, and frequent calcification, which may ultimately contribute to endodontic failure. Accordingly, the present study evaluated four diagnostic approaches, such as DVM-NT, DVM-T, DOM-NT, and DOM-T, to clarify the individual and combined effects of magnification and ultrasonic troughing on MB2 canal detection.

The results of this study demonstrated that MB2 canal detection rates were influenced by the detection strategy employed. The use of magnification was associated with higher MB2 detection rates; however, statistically significant superiority over DVMs was observed primarily when magnification was combined with ultrasonic troughing. This finding suggests that although enhanced illumination and magnification improve visualization of pulpal floor anatomy, magnification alone may not consistently overcome anatomical barriers such as calcified dentin shelves or obscured canal orifices. Interestingly, no statistically significant difference was observed between the DOM-NT and DVM-T groups. This finding suggests that enhanced visualization alone may provide a diagnostic benefit comparable to that of ultrasonic troughing performed without magnification, highlighting that neither magnification nor troughing is universally superior when applied in isolation, as also suggested by previous clinical findings [18].

The visual advantages provided by the DOM facilitate identification of subtle anatomical landmarks, including developmental grooves, isthmuses, and canal orifices, particularly in teeth with complex root canal configurations [19]. Given that most MB2 canals are narrow, curved, and frequently concealed beneath secondary dentin, improved visualization supports more accurate localization and negotiation of these canals [20]. Nevertheless, the present findings indicate that visualization alone may be insufficient in cases where dentinal obstructions limit access to the canal orifice.

In this context, ultrasonic troughing independently contributed to improved MB2 canal detection by enabling selective and controlled dentin removal along the pulpal floor. Compared with rotary burs, ultrasonic instruments provide greater tactile feedback and precision, particularly in areas of calcification or anatomical constriction [15,21]. When performed under magnification, ultrasonic troughing appears to maximize diagnostic efficiency by exposing hidden canal orifices while minimizing unnecessary dentin removal. This synergistic interaction between magnification and troughing was most evident in the DOM-T group, which demonstrated the highest detection rate among all experimental conditions.

A notable strength of this study lies in its two-factor experimental design combined with multivariable binary logistic regression analysis. Unlike previous investigations that assessed magnification or troughing in isolation or employed sequential designs, the present approach allowed the independent contributions of magnification and ultrasonic troughing to be quantified within the same statistical model. Magnification increased the odds of MB2 detection by nearly fourfold, while ultrasonic troughing increased detection odds by more than twofold. These findings indicate that the observed improvements are attributable to the distinct diagnostic contributions of each technique rather than to procedural sequencing or operator persistence.

The analysis of troughing depth further provides clinically relevant insights. In the DOM-T group, a greater proportion of MB2 canals were detected with a troughing depth of 2 mm or less compared with the DVM-T group. This observation suggests that magnification facilitates earlier recognition of canal anatomy, thereby limiting the extent of dentin removal required for canal localization. From a clinical standpoint, this finding aligns with the principles of minimally invasive endodontics [15], as excessive dentin removal may increase the risk of perforation and compromise long-term tooth integrity. Clinically, this suggests that the use of magnification may allow earlier identification of the MB2 canal, thereby reducing the need for deeper troughing and minimizing unnecessary dentin removal.

Previous studies have reported similar trends regarding the benefits of magnification and ultrasonic instrumentation in MB2 canal detection. Investigations comparing the DVM with magnification-assisted techniques have consistently demonstrated higher detection rates when enhanced visualization and selective dentin removal are employed. However, MB2 canal detection is influenced not only by diagnostic tools but also by access cavity design. Saygili et al. [14] reported that conservative and traditional endodontic access cavities resulted in higher MB2 detection rates compared with point access cavities, emphasizing the importance of adequate access geometry for canal localization. In their study, ultrasonic instruments were used as adjunctive tools, particularly in minimally prepared cavities, to facilitate MB2 detection. Nevertheless, despite the use of magnification and ultrasonic troughing, certain MB2 canals could only be identified using cone beam CT (CBCT), underscoring that even advanced clinical techniques may not reliably detect all canal anatomies. These observations support the present findings that ultrasonic troughing and magnification enhance MB2 detection but should be regarded as complementary strategies rather than definitive solutions.

Despite the demonstrated contribution of ultrasonic troughing to MB2 canal detection, its clinical application is not without limitations. Effective use of ultrasonic instruments requires specific equipment and an associated learning curve, which may limit widespread adoption, particularly among less experienced clinicians. Moreover, although ultrasonic techniques facilitate canal localization, they are not infallible and must be integrated with the clinician’s anatomical knowledge, tactile perception, and clinical experience. Accordingly, ultrasonic troughing should be considered an adjunctive tool rather than a standalone solution [22].

This study has several limitations. All procedures were performed by a single experienced operator, which may limit generalizability and introduce operator-related bias. However, this design minimized inter-operator variability and allowed consistent evaluation of the diagnostic contribution of each technique. Furthermore, the in vitro nature of the study precluded simulation of clinical factors such as bleeding, pulp tissue remnants, and operator fatigue. Nevertheless, the controlled experimental setting enabled isolation of the effects of magnification and ultrasonic troughing without confounding clinical variables, while the use of root sectioning as a reference standard provided a reliable assessment of true canal presence. Additionally, although CBCT may provide supplementary anatomical information, root sectioning was preferred as a direct and reliable reference standard for confirming the true presence of the MB2 canal.

Future in vivo studies should evaluate whether similar detection patterns occur under clinical conditions and assess the influence of operator experience, canal morphology, and adjunctive imaging modalities such as CBCT. Such investigations may further refine clinical strategies for MB2 canal localization and support evidence-based decision-making in endodontic practice.

## Conclusions

Within the limitations of this in vitro study, both microscopic magnification and ultrasonic troughing were independently associated with improved detection of the MB2 canal in maxillary first molars. The highest MB2 detection rate was achieved when these techniques were used in combination. Incorporating magnification and selective ultrasonic troughing into endodontic practice may improve the thoroughness of canal identification during root canal treatment. Further clinical studies are required to confirm these findings under in vivo conditions.
